# The Artificial Production of Organic Forms

**Published:** 1882-05

**Authors:** 


### The Artificial Production of Organic Forms.

The mimicry of organic forms by inorganic elements has often excited the attention of the curious, and it has been recently systematically studied, so far as chemical elements are concerned, by MM. Monnier and Vogt. Forms which closely resemble those of living organism, such as simple and tubercular cells, with septa and granular contents, may be artificially produced in a suitable liquid by the union of two salts, which form, by double decomposition, one or two insoluble compounds. One of the salts must be dissolved in the liquid, the other solid. These objects are formed equally well, whether one of the liquids is of organic origin (as sucrate of lime) or purely inorganic (as of silicate of soda). The resulting form clearly depends on the nature and viscid consistence and concentration of the liquids. Some viscid solutions, however, such as gum arabic and chloride of zinc, do not give rise to them. The form which results is as constant for the same conditions as is the crystalline form of a mineral, and it may even be employed as a means of ascertaining the presence of a given substance. It promises to constitute a new and very sensitive form of analysis, and may thus be employed, for instance, to distinguish the alkaline carbonates, sesquicarbonates, and bicarbonates, the one from the other. The chief influence which determines the form, is the nature of the acid which enters into the composition of the solid salt. The sulphates and phosphates, under certain circumstances, give rise to tubular forms, the carbonates to simple cells. With certain exceptions, such as the sulphates of copper, of cadmium, of nickel, and of zinc, these pseudo-organic forms are only produced by the combination of those substances which are found in real organisms. Sucrate of lime, for instance, give rise to such forms, while sucrate of strontia and of baryta do not. The pseudo-organic elements are surrounded by true membranes of perfect dialyzing power, allowing only liquids to pass. Their contents are heterogenous, and granules in the interior may be disposed in a special manner. They thus resemble absolutely the morphological elements of organized beings. It is highly probable that the inorganic constituents of living protoplasm play some part in determining the forms which the organized elements assume. —*London Lancet.*

